# The three-dimensional ordered macroporous structure of the Pt/TiO_2_–ZrO_2_ composite enhanced its photocatalytic performance for the photodegradation and photolysis of water

**DOI:** 10.1039/c8ra00998h

**Published:** 2018-05-23

**Authors:** Mingze An, Li Li, Yu Tian, Hualiang Yu, Qianlong Zhou

**Affiliations:** College of Materials Science and Engineering, Qiqihar University Qiqihar 161006 P. R. China qqhrll@163.com qqhrlili@126.com +86 04522738206; College of Chemistry and Chemical Engineering, Qiqihar University Qiqihar 161006 P. R. China

## Abstract

Using polystyrene (PS) spheres as a template, three-dimensional ordered macroporous Pt/TiO_2_–ZrO_2_ (3DOM Pt/TiO_2_–ZrO_2_) composites were prepared by vacuum impregnation combined with photoreduction. The crystal structure, composition, morphology, optical absorption, and surface physicochemical properties of the as-synthetized samples were characterized by X-ray diffraction (XRD), UV-visible diffuse reflectance spectroscopy (UV-vis/DRS), X-ray photoelectron spectroscopy (XPS), scanning electron microscopy (SEM), and N_2_ adsorption–desorption analyses. The results showed that the 3DOM Pt/TiO_2_–ZrO_2_ composites were mainly composed of anatase TiO_2_ and tetragonal ZrO_2_ crystal phases, in which Pt mainly existed as a single species. In addition, the as-synthesized composites had open, three-dimensionally ordered macroporous structures that could enhance their multi-mode photocatalytic degradation performance under UV, visible light, simulated solar light, and microwave-assisted irradiation. Moreover, the 3DOM Pt/TiO_2_–ZrO_2_ composites exhibited the best photocatalytic water splitting performance as compared to other systems.

## Abstract 2

Using polystyrene spheres as templates, 3DOM Pt/TiO_2_–ZrO_2_ composites were prepared by the vacuum impregnation combined with photoreduction method, which exhibited an enhanced photodegradation and water splitting performance.

## Graphical abstract

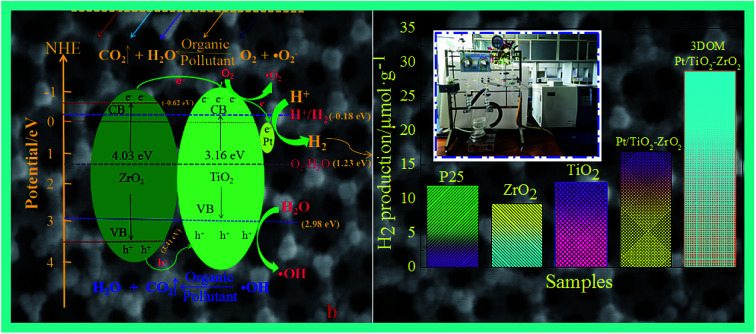

## 1 Introduction

A study on semiconductor photoelectrochemistry was published in Nature by Fujishima and Honda in 1972.^1^ The concept of exciting TiO_2_ semiconductors with UV light to split water has now become the basis for modern solar fuel research,^2^ and research on photocatalytic materials that are beneficial to human beings in the management of environmental pollution and improvement of living space has attracted significant attention.^3^

At present, TiO_2_ is a more widely used semiconductor photocatalyst with high photocatalytic efficiency and chemical stability and low cost; however, the recombination of photogenerated electron–hole pairs and the low quantum efficiency have limited the practical application of single semiconductor photocatalysts, such as TiO_2_, to some extent. Therefore, research on improving the photocatalytic efficiency of composite materials *via* the synergistic effect of the combination of a single semiconductor photocatalyst, such as TiO_2_, with other single semiconductor photocatalysts is increasing. The optical absorption range of TiO_2_ monomer is less than 400 nm, and the quantum efficiency is low.^4^ The combination of appropriate semiconductor materials and TiO_2_ can use the synergistic effect to further enhance the photocatalytic activity of TiO_2_. ZrO_2_ is an oxide with a wide bandgap (*ca.* 3–5 eV). Moreover, the surface of ZrO_2_ has both acidic and basic properties; therefore, ZrO_2_ exhibits oxidation and reduction properties and shows photocatalytic activity.^5,6^ ZrO_2_ is a typical p-type semiconductor with three crystal forms: it is monoclinic at room temperature and undergoes a tetragonal phase transition with a change in temperature. The tetragonal phase has high catalytic activity and has been extensively studied.^7,8^

In recent years, researchers have often improved the photocatalytic performance of composites by various methods during the synthesis process.^9^ For the first time, Velev *et al.* used polystyrene colloidal microspheres as templates to prepare ordered macroporous SiO_2_.^10^ Because the preparation process is relatively simple and the pore structure is easy to be controlled, this approach has been developed as one of the most popular preparation methods to achieve three-dimensional ordered macroporous composites; Stein *et al.* have used 3DOM α-Al_2_O_3_ as a support for metallic silver in the ethylene epoxidation reaction and found that the catalytic efficiency of the 3DOM α-Al_2_O_3_ support is significantly higher than that of the commercial α-Al_2_O_3_ support.^11^ Thus, studies on the use of polystyrene microspheres as templates to prepare composites have shown that a three-dimensional ordered macroporous structure is excellent to the reaction process of reactants and product molecules into and out, which can increase the reactive sites and improve the catalytic activity to a certain extent.

In addition, the modification of semiconductors with precious metals has attracted significant attention. The change in the electron distribution in the system affects the surface properties of the semiconductor and can improve its photocatalytic activity; studies on the deposition of noble metals, including Ag, Pt, Rh, Pd, Ru, Au, Ir, *etc.*, have been reported, and studies in which Pt is used are more common because the modification effect of Pt is best as compared to that of other noble metals.^12–15^ The photocatalytic water splitting production of Pt/TiO_2_ could reach 33.0 mmol after 6 h of light irradiation, as reported by Zou *et al.*^16^ Therefore, high-activity materials for photocatalytic hydrogen production are expected to be prepared by doping Pt into semiconductor TiO_2_ and ZrO_2_ composites.^17–19^

Based on the existing experimental studies, polystyrene (PS) spheres were used as templates to prepare three-dimensionally ordered macroporous Pt/TiO_2_–ZrO_2_ composites by photoreduction. On the one hand, the noble metal Pt was supported as an electron trap to suppress the recombination of photogenerated electron–hole pairs in the semiconductor TiO_2_ and ZrO_2_ to effectively capture the photogenerated electrons and promote the separation of photogenerated electron–hole pairs. Moreover, the captured photogenerated electrons can be better involved in the reaction of hydrogen production. On the other hand, the characteristics, such as single pore size, uniform pore size distribution, and orderly arrangement, of three-dimensional ordered macroporous materials endow 3D porous materials with higher porosity, larger specific surface area, and stronger surface adsorption capacity, which can effectively improve the photocatalytic activity of composite materials.

In this study, the photocatalytic degradation of 3DOM Pt/TiO_2_–ZrO_2_ composites was investigated by multi-mode photocatalytic experiments. Moreover, the photocatalytic performance of the composites was further investigated. The experimental result indicated that the three-dimensional ordered macroporous structure of the composite material enhanced the photocatalytic performance.

## 2 Experimental

### 2.1 Materials

Titanium tetraisopropoxide (TTIP, purity 98%) was purchased from New Jersey, USA; zirconium *n*-butoxide (C_16_H_36_O_4_Zr) was purchased from Shanghai Chunhe Biotechnology Co., Ltd.; chloroplatinic acid was purchased from Shanghai Jingjing Biochemical Technology Co., Ltd.; tri-block copolymer (P123) was purchased from Aldrich Company in the United States; styrene, commercial photocatalyst (Degussa P25), K_2_S_2_O_8_, methyl orange (MO), congo red (CR), methylene blue (MB), and salicylic acid (SA) were purchased from Tianjin Mitsufu Fine Chemicals Research Institute. All chemicals were of analytical grade (AR) and used as received without purification; secondary distilled water was employed for all the experiments.

### 2.2 Preparation of 3DOM Pt/TiO_2_–ZrO_2_

(a) At first, 2.0 g of polystyrene (PS) spheres synthesized by the non-emulsification polymerization technology reported in the literature^20^ was impregnated with 8 mL methanol, stirred for 30 min, then filtered, and naturally dried. Then, *n*-butoxide and titanium tetraisopropoxide were mixed uniformly at a volume ratio of 1 : 4. The treated PS spheres were then slowly added, stirred for 1 h, and filled in under vacuum. The resulting solid mixture was calcined for 8 h to obtain the composites marked as 3DOM TiO_2_–ZrO_2_.

(b) The 3DOM TiO_2_–ZrO_2_ composites were placed in 50 mL of deionized water and sonicated for 10 min. Then, 3.0 g of sodium sulfide was added as a sacrificial agent in the photoreduction reactor, and 0.15 mL of platinum standard solution was added. The mixed solution was then irradiated for 1 h using a 300 W xenon lamp for light reduction of Pt, washed four times with deionized water and ethanol, and vacuum dried to obtain the composites marked as 3DOM Pt/TiO_2_–ZrO_2_.

(c) Typically, 14 mL of ethanol and 2 mL of isopropanol were added into a 50 mL beaker, and titanium tetraisopropoxide and zirconium *n*-butoxide at a volume ratio of 1 : 4 were added. The mixture was continuously stirred at room temperature, and a small amount of deionized water (*ca.* 0.5 mL) was added until a gel-like material appeared. The resulting gel was dried for 12 h. Finally, the resulting solid was calcined at 600 °C for 8 h to obtain the desired nanocomposites TiO_2_–ZrO_2_.

(d) The Pt/TiO_2_–ZrO_2_ composites used for comparative analysis were prepared by hydrothermal synthesis and photoreduction with the same ratio of feedstock. The other monomers were prepared under the same experimental conditions.

### 2.3 Characterization

The phase and composition of the as-prepared samples were determined using a Bruker-AXS (D8) X-ray diffractometer (XRD) equipped with Cu Kα as the X-ray radiation source at 60 kV, 80 mA, and 2*θ* ranging from 20° to 80°; X-ray photoelectron spectroscopy (XPS) was performed using an ESCALAB 250Xi spectrometer equipped with an Al Kα radiation source at 300 W. SEM analysis of the samples was carried out using a Japanese Hitachi S-4700 scanning electron microscope, and the working voltage was 5 kV. The specific surface area and pore size of the samples were measured using a specific surface area instrument (Beishide Instrumentation Technologies Ltd., Model 3H-2000PS2, Beijing, China) involving nitrogen adsorption at 77 K. The UV-vis absorption spectra were obtained using a UV-vis spectrophotometer (Model TU-1901) in the wavelength range of 200–800 nm, and BaSO_4_ was used as a reference. The absorbance of the sample solution was determined using a TU-1901 UV-visible double-beam spectrophotometer (Beijing Purkinje General Co.). The photolysis of water to hydrogen was measured using a Lab-Solar-IIIAG photocatalytic line analysis system (Perfect Light Ltd., Beijing, China). Photocatalytic hydrogen production was analyzed *via* a GC7900 gas chromatograph (Tianmei Instrument Co., Ltd., Shanghai) operating at a voltage of 50 mV and a current of 20 mA using the external standard method.

### 2.4 Photocatalytic tests

The photocatalytic experimental reactors under different modes were self-made. The UV light source was a 125 W high-pressure mercury lamp (the main emission wavelength was 313.2 nm), the visible light source was a 400 W xenon lamp (the main emission wavelength was greater than 410 nm), and the simulated daylight light source was a 1000 W xenon lamp (external type, Shanghai Bao Xun Instrument Co., Ltd., the emission spectrum was close to the full spectrum). The microwave photocatalytic experiments used H-type microwave electrodeless lamps as the light source with a power of 15 W and a microwave reactor output power of 600 W; details about the microwave photocatalytic reactor can be found in the literature.^21^ In the experiments, 0.15 g, 0.3 g, 0.15 g, and 0.5 g of catalyst were dispersed in the newly prepared solution (concentration of 50 mg L^−1^), and the volume was 90 mL, 220 mL, 100 mL, and 500 mL, respectively. The suspension was sonicated for 10 min, stirred in the dark for 30 min, and then placed in a photocatalytic reactor for photocatalytic experiments. Samples were obtained at regular intervals during the reaction, and their absorbance was measured at *λ*_max_ by a UV-vis spectrophotometer.

## 3 Results and discussion

### 3.1 XRD analysis

**Fig. 1:**
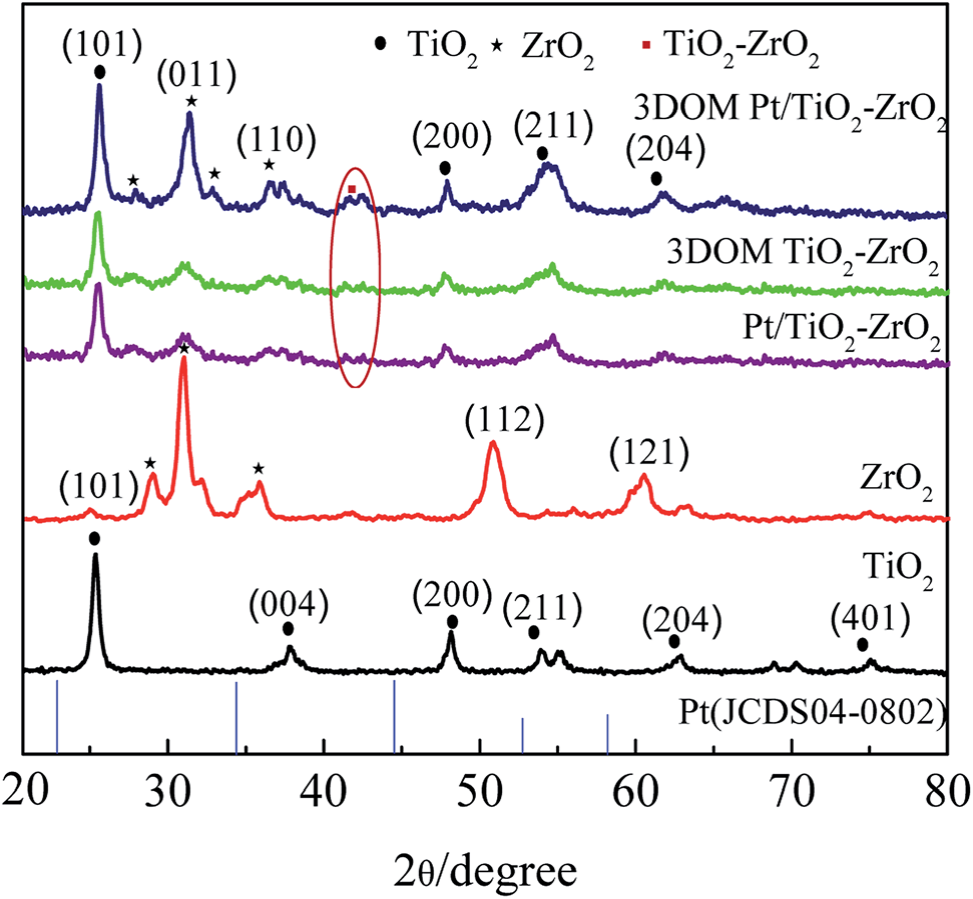
X-ray diffraction patterns of different materials.

To investigate the crystal structure of 3DOM Pt/TiO_2_–ZrO_2_, X-ray diffraction analysis of 3DOM Pt/TiO_2_–ZrO_2_, 3DOM TiO_2_–ZrO_2_, monomer TiO_2_, and ZrO_2_ was carried out, and the results are shown in Fig. 1. The main peaks of monomer TiO_2_ located at 25.36°, 39.77°, 48.11°, 53.90°, 62.87°, and 75.20° belong to the anatase phase, corresponding to the crystal planes of (101), (004), (200), (211), (204) and (401) (JCPDS 17-0923).^22^ Monolithic ZrO_2_ has a tetragonal crystal phase, and the crystal planes of the main diffraction peaks at 31.06°, 36.10°, 50.90°, and 60.55° belong to (011), (110), (112), and (121), respectively (JCPDS 17-0923).^23^ The diffraction peaks of Pt/TiO_2_–ZrO_2_ are mainly located at 25.36°, 31.06°, 36.10°, 48.11°, 53.90°, and 62.87°, corresponding to the (101), (011), (110), (211), (204) and (401) crystal planes. The diffraction peaks of 3DOM TiO_2_–ZrO_2_ in the three-dimensional ordered macroporous composites are mainly located at 25.36°, 31.06°, 36.10°, 48.11°, 53.90°, and 62.87°. The diffraction peak of the labelled ring in Fig. 1 corresponds to the weaker TiO_2_–ZrO_2_ diffraction peak, indicating the presence of Ti–O–Zr bonds in the composites.^24^ The diffraction peaks of 3DOM Pt/TiO_2_–ZrO_2_ in the three-dimensionally ordered composites are mainly located at 25.36°, 31.06°, 36.10°, 48.11°, 53.90°, and 62.87°. The characteristic peak of Pt does not appear because of the small doping amount of Pt. The abovementioned results show that the 3DOM TiO_2_–ZrO_2_ and 3DOM Pt/TiO_2_–ZrO_2_ composites mainly consist of a TiO_2_ anatase phase and ZrO_2_ tetragonal phase.^25^

The average crystallite size of each synthesized product was calculated according to the Scherrer formula *d* = *Kλ*/(*B* cos *θ*). The results are shown in Table 1, where *d* is the average grain diameter of the crystal grains, *K* is a constant (0.89), *B* is the diffraction peak half-width, *λ* is the X-ray wavelength of 0.154178 nm, and *θ* is the Bragg angle corresponding to the diffraction peak.^26^ As seen in Table 1, the crystallite size of the composites is smaller than that of pure ZrO_2_ and TiO_2_. Moreover, the incorporation of Pt can affect the crystallite size of the composites to a certain extent. As can be seen from the data presented in Table 1, the crystallite size of the 3DOM Pt/TiO_2_–ZrO_2_ composites is significantly larger than that of the 3DOM TiO_2_–ZrO_2_ composites; this is attributed to the lattice distortion of the composite material due to Pt doping. Moreover, the unit cell parameters further prove that the grain size increases with Pt doping.

**Table 1:** Unit cell parameters (Å), grain size (*d*), and energy bandgap (*E*_g_) of each sample

Sample	Unit cell parameters/Å			*d*/nm	*E*_g_/(eV)
	*a*/Å	*b*/Å	*c*/Å		
ZrO_2_	3.596	3.596	5.184	47.5	4.03
TiO_2_	3.254	3.254	4.568	32.6	3.16
3DOM TiO_2_–ZrO_2_	2.856	2.856	4.239	21.8	3.25
3DOM Pt/TiO_2_–ZrO_2_	2.994	2.994	4.437	25.6	3.02

### 3.2 UV-visible diffuse reflectance spectroscopy

**Fig. 2:**
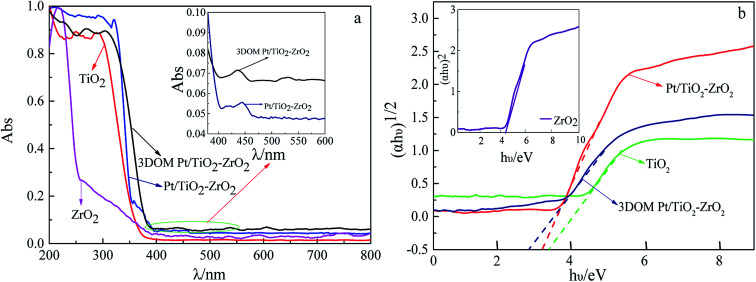
UV-vis diffuse reflectance absorption spectra (a) and Kubelka–Munk energy plots (b) of different materials.

To understand the optical absorption properties of 3DOM Pt/TiO_2_–ZrO_2_, UV-vis diffuse reflectance spectroscopy of the monomer TiO_2_ and ZrO_2_ and Pt/TiO_2_–ZrO_2_ and 3DOM Pt/TiO_2_–ZrO_2_ composites was carried out, as shown in Fig. 2. The absorption performance of 3DOM Pt/TiO_2_–ZrO_2_ in the ultraviolet region is enhanced as compared to that of TiO_2_ and ZrO_2_; this indicates that the incorporation of Pt changes the optical absorption properties of TiO_2_ and ZrO_2_. Moreover, compared with that of the Pt/TiO_2_–ZrO_2_ composites, the absorption of 3DOM Pt/TiO_2_–ZrO_2_ composites is higher, and some redshifts occur in the UV region, indicating that the three-dimensional ordered macroporous structure can further enhance the absorption of the material. However, the visible light absorption does not increase upon Pt doping; this should be attributed to the small doping amount of Pt.

The bandgap energies of TiO_2_, ZrO_2_, Pt/TiO_2_–ZrO_2_, and 3DOM Pt/TiO_2_–ZrO_2_ were obtained according to Fig. 2(b) and the Kubelka–Munk formula:^27^*αhν* = *A*(*hν* − *E*_g_)^*n*/2^where *α* is the absorption coefficient, *n* is the optical frequency, and *A* is the proportionality constant. As obtained from the abovementioned equation, the *E*_g_ values of TiO_2_, ZrO_2_, Pt/TiO_2_–ZrO_2_, and 3DOM Pt/TiO_2_–ZrO_2_ are 3.16 eV, 4.03 eV, 3.56 eV, and 3.02 eV, respectively. From the as-obtained results, it can be seen that the *E*_g_ value of 3DOM Pt/TiO_2_–ZrO_2_ decreases obviously as compared to that of TiO_2_, ZrO_2_, and the composite Pt/TiO_2_–ZrO_2_. To a certain extent, it indicates that the Pt/TiO_2_–ZrO_2_ composites with a three-dimensional ordered macroporous structure will show higher photocatalytic activity.

### 3.3 XPS analysis

**Fig. 3:**
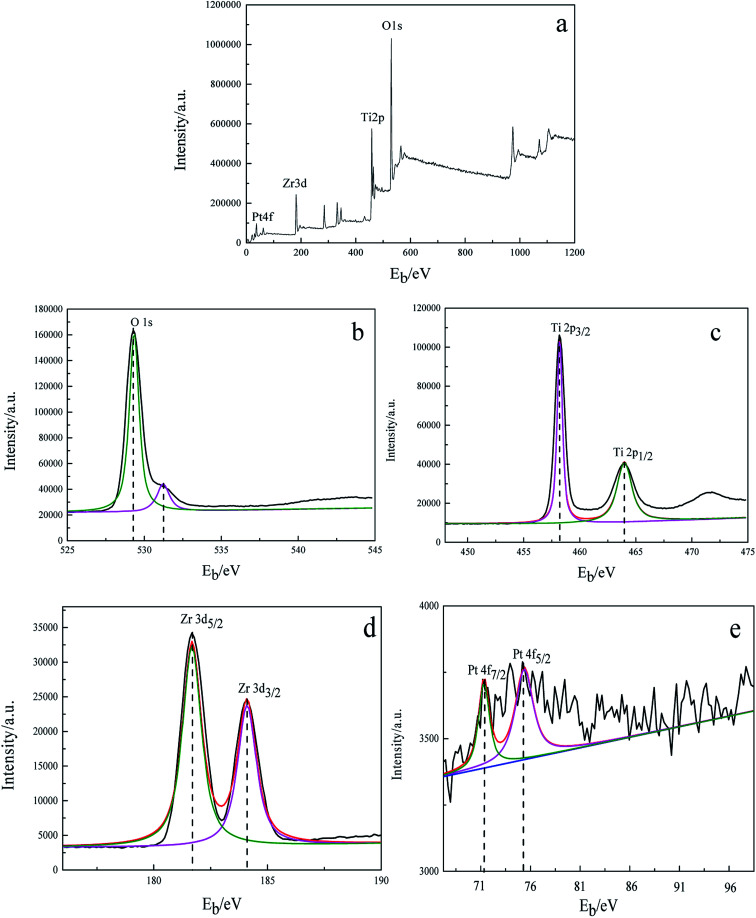
X-ray photoelectron spectra of 3DOM Pt/TiO_2_–ZrO_2_. (a) Full spectrum; (b) O 1s; (c) Ti 2p; (d) Zr 3d; and (e) Pt 4f.

To study the valence of the surface elements of 3DOM Pt/TiO_2_–ZrO_2_ composites, XPS analysis was carried out. It can be seen from Fig. 3a that there are four elements, *i.e.* Pt, Zr, Ti and O, on the surface of the 3DOM Pt/TiO_2_–ZrO_2_ composites. Fig. 3b shows the XPS spectrum of O 1s in the composites with the binding energies of 527.27 eV and 531.25 eV, indicating the presence of lattice oxygen and adsorbed oxygen, respectively.^28^Fig. 3c shows the XPS spectrum of Ti in the Ti 2p_3/2_ and Ti 2p_1/2_ binding energy regions, and the binding energy is 458.25 eV and 463.75 eV, respectively, indicating that Ti is in the Ti^4+^ form.^29^Fig. 3d shows the XPS spectrum of Zr in the Zr 3d_5/2_ and Zr 3d_3/2_ binding energy regions, with the binding energies of 181.69 and 184.08 eV, respectively, indicating that Zr is in the Zr^4+^ form.^30^Fig. 3e shows the XPS spectrum of Pt in Pt 4f_7/2_ and Pt 3d_5/2_ in the composites, with the binding energies of 71.51 eV and 75.45 eV, respectively, indicating that Pt exists as a single species.^31^

### 3.4 SEM analysis

**Fig. 4:**
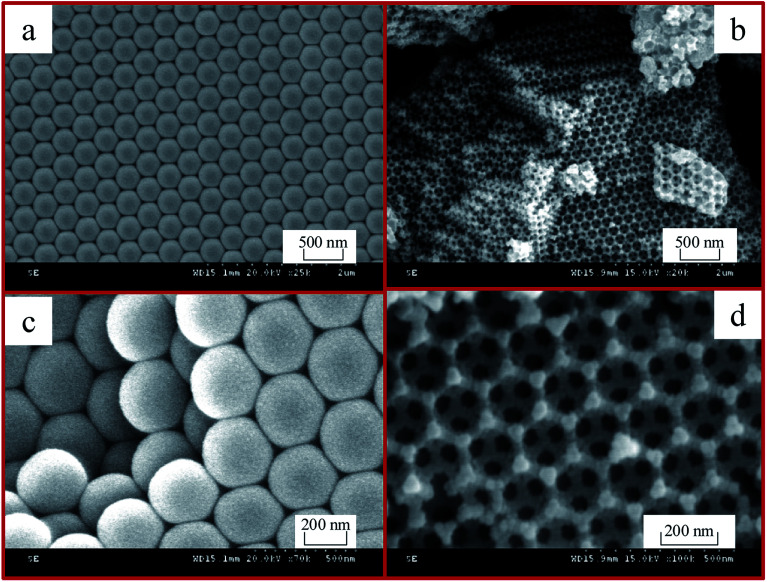
SEM images of polystyrene (PS) microspheres (a and c) and 3DOM Pt/TiO_2_–ZrO_2_ (b and d).

Fig. 4 shows the SEM images of the polystyrene (PS) microspheres and 3DOM Pt/TiO_2_–ZrO_2_ composites. It can be seen in Fig. 4(a) and (c) that the PS spheres are very homogeneous and exhibit a monodisperse and closely packed structure with an average diameter of *ca.* 300 nm. Fig. 4(b) and (d) show the SEM images of 3DOM Pt/TiO_2_–ZrO_2_ calcined after the removal of polystyrene (PS) spheres under different scales. We can see from Fig. 4(b) that the 3DOM Pt/TiO_2_–ZrO_2_ composites have a neat and ordered arrangement, and the size of the macropores is basically the same, with an average diameter of about 200 nm. The hole wall is composed of nanocrystalline Pt/TiO_2_–ZrO_2_. In addition, a small number of defects in some areas are related to the arrangement of the PS microspheres, and the size of the microspheres depends mainly on the diameter of the polystyrene (PS) sphere template. It can be observed from Fig. 4 that the pore size of 3DOM Pt/TiO_2_–ZrO_2_ is much smaller than that of the polystyrene (PS) colloidal spheres; this is due to the shrinkage of pores during the calcination process for removing the polystyrene (PS) colloidal spheres.

### 3.5 N_2_ adsorption–desorption analysis

**Fig. 5:**
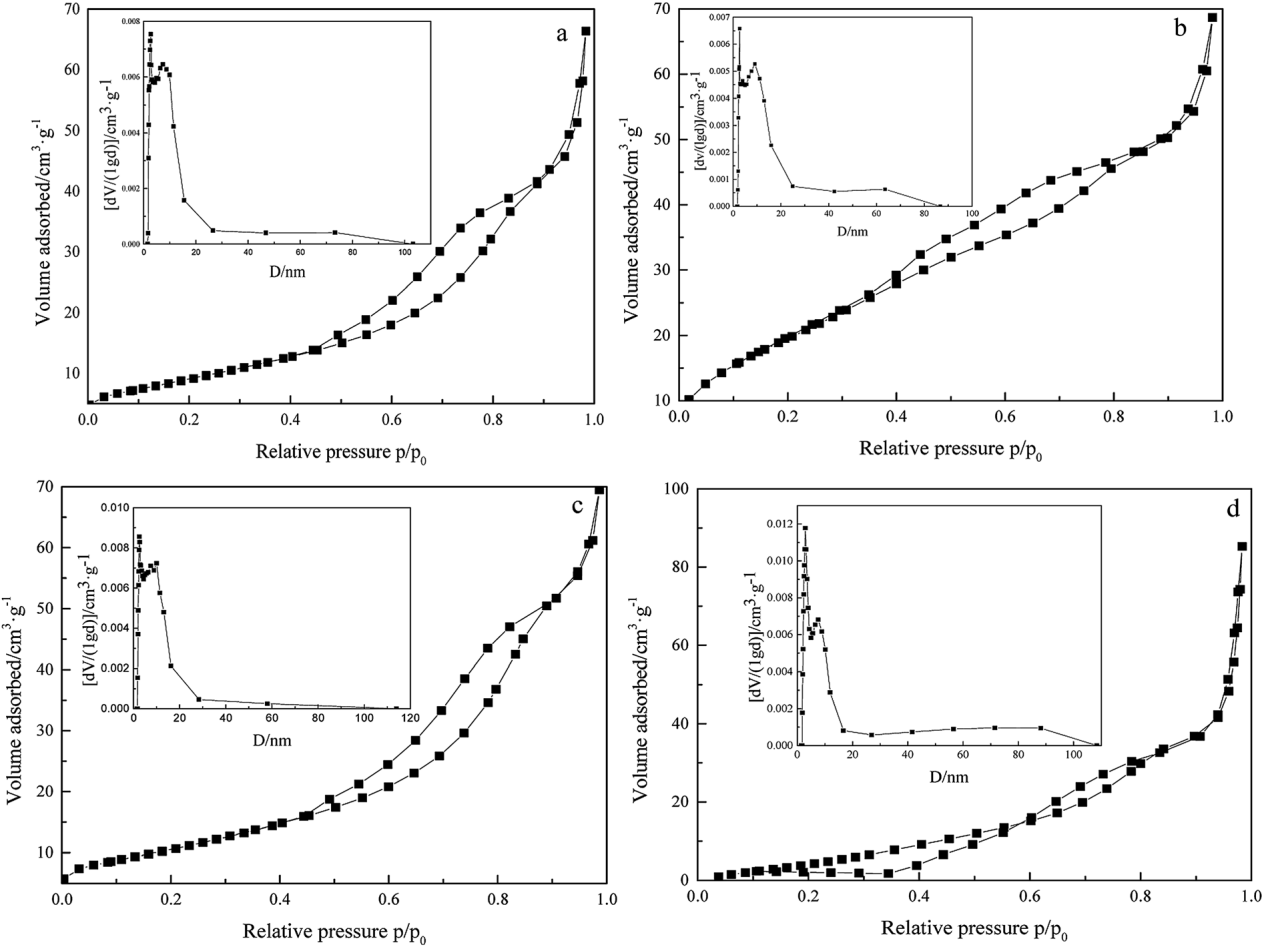
N_2_ adsorption–desorption isotherms of 3DOM Pt/TiO_2_–ZrO_2_ (a), Pt/TiO_2_–ZrO_2_ (b), 3DOM TiO_2_–ZrO_2_ (c), and TiO_2_–ZrO_2_ (d) (insets show the BJH pore size distribution curves).

To study the surface physicochemical properties of 3DOM Pt/TiO_2_–ZrO_2_ composites, N_2_ adsorption–desorption tests for Pt/TiO_2_–ZrO_2_, 3DOM TiO_2_–ZrO_2_, TiO_2_–ZrO_2_, and 3DOM Pt/TiO_2_–ZrO_2_ were carried out, as shown in Fig. 5 and Table 2. As seen in Fig. 5, the N_2_ adsorption–desorption isotherms of Pt/TiO_2_–ZrO_2_, 3DOM TiO_2_–ZrO_2_, TiO_2_–ZrO_2_, and 3DOM Pt/TiO_2_–ZrO_2_ are type IV adsorption curves.^32^ According to the IUPAC definition, they have typical mesoporous structures. The hysteresis loop of the Pt/TiO_2_–ZrO_2_ composites belongs to H2 type, and the hysteresis loop of this kind of materials is characterized by the straight hole model. Moreover, the hysteresis loop of 3DOM Pt/TiO_2_–ZrO_2_ and 3DOM TiO_2_–ZrO_2_ is of H3 type and caused by the aggregation of the capillary and the aggregation of the particles in the structure. In addition, the hysteresis loop of TiO_2_–ZrO_2_ belongs to the bicyclic structure of H4 type.^33^ 3DOM Pt/TiO_2_–ZrO_2_ shows a mesoporous structure mainly due to the macroporous wall of the composites. It can be seen from Fig. 5a and d that the structure is different mainly because the composites shown in Fig. 5a have been synthesized using polystyrene spheres as templates and form a three-dimensionally ordered macroporous structure, whereas the composites shown in Fig. 5d are composed of TiO_2_ and ZrO_2_, which do not have a three-dimensionally ordered structure.

**Table 2:** Specific surface area, average pore size, and pore volume of Pt/TiO_2_–ZrO_2_, TiO_2_–ZrO_2_, 3DOM TiO_2_–ZrO_2_, and 3DOM Pt/TiO_2_–ZrO_2_

Sample	*S*_BET_/m^2^ g^−1^	Pore diameter/nm	Pore volume/cm^3^ g^−1^
3DOM Pt/TiO_2_–ZrO_2_	34.25	12.00	0.1027
3DOM TiO_2_–ZrO_2_	39.49	10.89	0.1075
Pt/TiO_2_–ZrO_2_	76.21	5.57	0.1062
TiO_2_–ZrO_2_	31.10	16.96	0.1319

As can be seen from Table 2, 3DOM Pt/TiO_2_–ZrO_2_ shows a certain decrease in the specific surface area (BET) as compared to 3DOM TiO_2_–ZrO_2_; this may be because the doping of Pt has blocked some large pores that results in a smaller surface area.

Compared with TiO_2_–ZrO_2_, Pt/TiO_2_–ZrO_2_ has a larger surface area; this may be because the presence of some amorphous structures in the material results in a larger surface area. In addition, the specific surface area of Pt/TiO_2_–ZrO_2_ composites is larger than that of 3DOM TiO_2_–ZrO_2_; this can be attributed to their different hysteresis loops and synthesis methods. Due to the use of the methanol vacuum impregnation method in the synthesis process, the precursors are very evenly filled within the pores of polystyrene microspheres, which can improve the photocatalytic activity of the composite materials.

### 3.6 Photocatalytic activity for the degradation of dyes

**Fig. 6:**
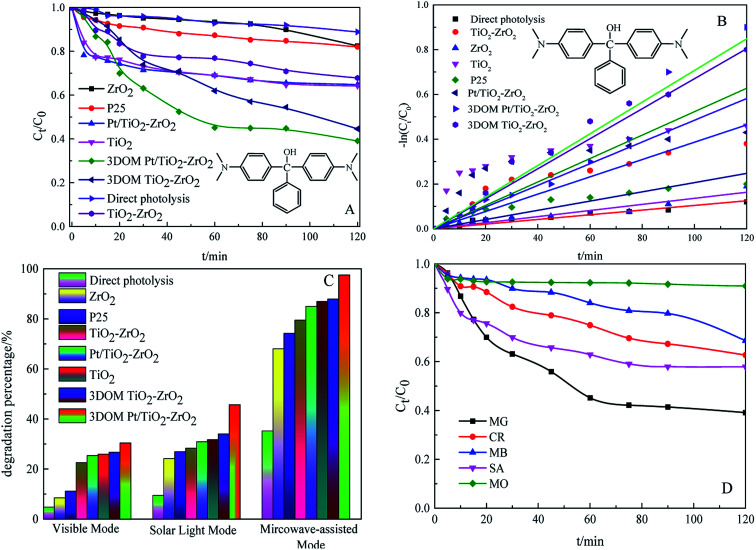
Photodegradation of malachite green with different catalysts under UV light irradiation (A), kinetics diagram of different catalysts for the degradation of malachite green (B), photodegradation of malachite green with different catalysts under simulated sunlight and microwave-assisted irradiation (C), and results of photodegradation of different dyes by 3DOM Pt/TiO_2_–ZrO_2_ (D).

To investigate the photocatalytic activity of 3DOM Pt/TiO_2_–ZrO_2_ composites, multi-mode photocatalytic experiments were carried out under UV light, visible light, simulated sunlight, and microwave-assisted irradiation. The results are shown in Fig. 6. It can be seen from Fig. 6A that the 3DOM Pt/TiO_2_–ZrO_2_ composites show the best degradation effect on the organic pollutant malachite green (MG) under ultraviolet light irradiation, and the catalytic activity is remarkably enhanced after the addition of Pt; this is similar to the result of the previous UV-visible diffuse reflectance absorption spectroscopy analysis. Due to the strong absorption capacity of monomer TiO_2_ and monomer ZrO_2_ in the ultraviolet region, the synergistic effect between Pt and TiO_2_ can be further improved by Pt doping. Upon comparing the results of direct degradation by different catalysts under UV light, it is found that 3DOM Pt/TiO_2_–ZrO_2_ exhibits increased photocatalytic activity for the degradation of malachite green under ultraviolet irradiation. From Fig. 6B, it is clear that −ln *C*_t_/*C*_0_ has a substantially linear relationship with the reaction time *t*; this indicates that the degradation of malachite green follows quasi-first order reaction kinetics. The catalytic activity order for dye degradation was 3DOM Pt/TiO_2_–ZrO_2_ > 3DOM TiO_2_–ZrO_2_ > TiO_2_ > Pt/TiO_2_–ZrO_2_ > TiO_2_–ZrO_2_ > P25 > ZrO_2_ > direct photolysis.

It can be seen from Fig. 6C that 3DOM Pt/TiO_2_–ZrO_2_ has the highest photocatalytic degradation rate of malachite green under visible light degradation, simulated sunlight degradation, and microwave-assisted degradation; this indicates that the three-dimensional ordered macroporous structure can enhance the photocatalytic activity to a certain extent. However, the photocatalytic activity of 3DOM Pt/TiO_2_–ZrO_2_ composites under visible light is far less than that under UV light because 3DOM Pt/TiO_2_–ZrO_2_ has little absorption in the visible region (380–800 nm); therefore, its photocatalytic activity in the visible region is not high. This result is consistent with the UV-vis diffuse reflectance spectroscopy analysis results. Moreover, compared with other catalytic materials, 3DOM Pt/TiO_2_–ZrO_2_ has better degradation effect on malachite green under the simulated sunlight condition. The abovementioned results show that the degradation effect of 3DOM Pt/TiO_2_–ZrO_2_ is further increased, and 3DOM Pt/TiO_2_–ZrO_2_ has more obvious practical application effects on the dye model molecule under simulated sunlight irradiation.

Finally, the catalytic activity of the three-dimensional ordered macroporous composites under microwave irradiation is higher than that under ultraviolet light, visible light, and simulated sunlight. This is partly because under the microwave-assisted condition, the used electrodeless lamp also emits ultraviolet light, but the lamp power (15 W) is smaller than that of the ultraviolet lamp (125 W). On the other hand, due to the good crystallinity and narrow bandgap of 3DOM Pt/TiO_2_–ZrO_2_, which can induce better activity, the macroporous structure can enhance the contact area with pollutants. More contact active sites further enhance the activity of the catalyst.

As can be seen in Fig. 6D, the 3DOM Pt/TiO_2_–ZrO_2_ composites show a certain degradation effect on malachite green, congo red, methylene blue, methyl orange, and salicylic acid within 120 min; this indicates that the 3DOM Pt/TiO_2_–ZrO_2_ composites display a certain degree of universality in the degradation of organic pollutants.

### 3.7 Photocatalytic hydrogen evolution

**Fig. 7:**
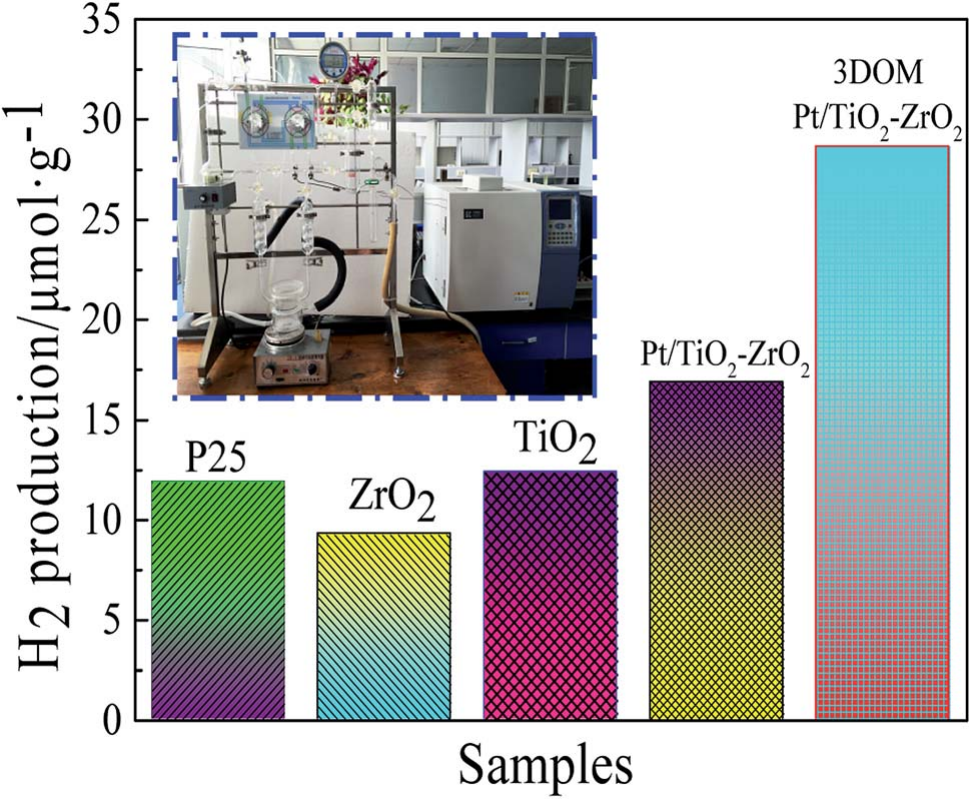
Hydrogen production amount for P25, ZrO_2_, TiO_2_, Pt/TiO_2_–ZrO_2_, and 3DOM Pt/TiO_2_–ZrO_2_ photocatalysts (*t* = 8 h).

As seen in Fig. 7, compared with P25, TiO_2_, ZrO_2_ and Pt/TiO_2_–ZrO_2_, 3DOM Pt/TiO_2_–ZrO_2_ possesses a certain ability in hydrogen production; this is attributed to the unique large-pore structure of 3DOM Pt/TiO_2_–ZrO_2_ that allows light to be internally reflected multiple times. Moreover, the neat and orderly large pore arrangement reduces light consumption and enhances the light absorption efficiency. According to the nitrogen adsorption–desorption test results, 3DOM Pt/TiO_2_–ZrO_2_ composites have a larger specific surface area to varying degrees that can improve the photocatalytic activity of the sample. Upon doping the noble metal Pt, the light absorption of the catalyst is improved. Moreover, the introduction of Pt increases the migration paths of the photogenerated carriers and reduces the recombination of photogenerated electron–hole pairs. In addition, Pt acts as a surface active site and increases the hydrogen production activity.

### 3.8 Possible photocatalytic reaction mechanism

**Fig. 8:**
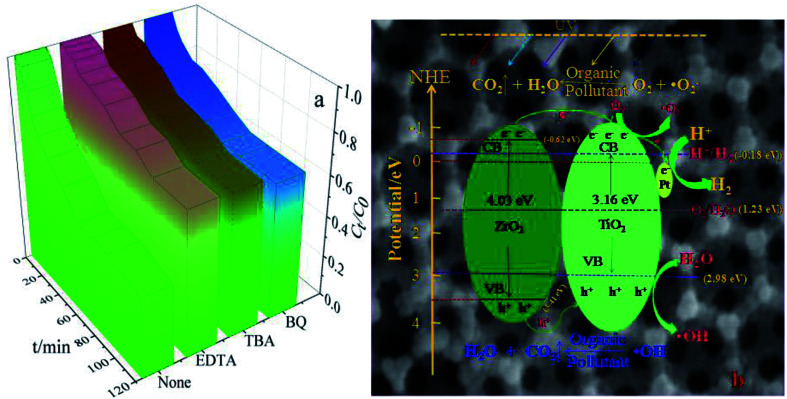
UV photocatalytic degradation of MG with different trapping agents (a) and possible photocatalytic reaction mechanism of 3DOM Pt/TiO_2_–ZrO_2_ (b).

As seen in Fig. 8a, the major active factor in the 3DOM Pt/TiO_2_–ZrO_2_ composite is (·OH), whereas superoxide radicals (·O_2_^−^) and holes (h^+^) play an auxiliary role. Based on the abovementioned results of the catalytic activity and related experimental data, a possible photocatalytic reaction mechanism of 3DOM Pt/TiO_2_–ZrO_2_ is speculated, as shown in Fig. 8b. On the one hand, the photocatalytic activity of 3DOM Pt/TiO_2_–ZrO_2_ can be improved due to the plasmon resonance effect on the surface of the noble metal Pt. On the other hand, when Pt nanoparticles are loaded on the n-type semiconductor TiO_2_ and the p-type semiconductor ZrO_2_, the work function (5.65 eV) of Pt has a higher bandgap energy than that of the semiconductor TiO_2_; therefore, a Schottky barrier is formed between them.^34^ Due to the Schottky barrier, when the photocatalytic reaction of TiO_2_ is conducted under light, the electrons from the separated electron–hole pairs aggregate on the contact surface of Pt and TiO_2_. Moreover, Pt can act as an electron trap, promoting the separation of electron–hole pairs and increasing the electron transfer between interfaces. The three-dimensional ordered macroporous structure provides a good channel for the transfer of the active material, thereby improving the photocatalytic activity of the material.^35^ The positions of the conduction band (CB) and valence band (VB) are calculated by the following formula:

1 *E*_CB_ = *χ* − *E*_e_ − 0.5*E*_g_

2 *E*_VB_ = *E*_c_ + *E*_g_

The redox potentials of the conduction band and the valence band of TiO_2_ are −0.18 eV and 2.98 eV, respectively. The oxidation–reduction potentials of the conduction band and the valence band of ZrO_2_ are −0.62 eV and 3.41 eV, respectively. This satisfies the conditions for the photocatalytic degradation of organic pollutants, and 3DOM Pt/TiO_2_–ZrO_2_ has the ability to photolyse water to produce hydrogen. As shown in Fig. 8b, the photoelectrons in the conduction band can react with oxygen adsorbed on the catalyst surface to generate superoxide radicals (·O_2_^−^); moreover, the holes (h^+^) remaining in the valence band can react with water to form hydroxyl radicals (·OH), which can degrade organic pollutants; in general semiconductor photocatalysts, the hydroxyl radicals do not easily contact with organic pollutants, whereas the three-dimensional ordered macroporous structure allows the organic pollutants to directly contact the hydroxyl radicals such that these pollutants can be degraded more effectively.

Moreover, the conduction band potential of 3DOM Pt/TiO_2_–ZrO_2_ composites is lower than that of the hydrogen electrode (0 eV), thus meeting the condition for hydrogen evolution, and 3DOM Pt/TiO_2_–ZrO_2_ can decompose water into hydrogen. Therefore, the 3DOM Pt/TiO_2_–ZrO_2_ composites can catalyze both the photocatalytic degradation and photolysis of water to produce hydrogen. On the one hand, the three-dimensional ordered macroporous structure can allow organic pollutants to directly contact the active material, thereby enhancing photocatalytic degradation. On the other hand, it can provide a convenient channel for the generated hydrogen and improve the efficiency of hydrogen evolution. Therefore, the three-dimensional ordered macroporous structure can enhance the photocatalytic activity of Pt/TiO_2_–ZrO_2_.

## 4 Conclusion

Herein, three-dimensional ordered macroporous Pt/TiO_2_–ZrO_2_ composites were prepared by vacuum impregnation combined with photoreduction using polystyrene (PS) spheres as a template synthesized *via* emulsion-free polymerization. The crystal structure of 3DOM Pt/TiO_2_–ZrO_2_ was better than that of other composites. Moreover, the composite material treated by the crystal template arranged in a neat and orderly manner, and the walls of the pores were formed by nanoparticle accumulation. Pt was present in the composite material as a simple substance, which could bring about some strong absorptions and redshifts in the ultraviolet region. The hydrogen production activity and photocatalytic degradation activity of the three-dimensional ordered macroporous Pt/TiO_2_–ZrO_2_ composites were higher than those of other systems; this indicated that the synergistic effect of the three materials and the unique three-dimensional ordered macroporous structure were conducive to improving the photolytic hydrogen production and photocatalytic degradation activity.

## Conflicts of interest

There are no conflicts to declare.

## Supplementary Material
